# Completeness of malaria indicator data reporting via the District Health Information Software 2 in Kenya, 2011–2015

**DOI:** 10.1186/s12936-017-1973-y

**Published:** 2017-08-17

**Authors:** Sophie Githinji, Robinson Oyando, Josephine Malinga, Waqo Ejersa, David Soti, Josea Rono, Robert W. Snow, Ann M. Buff, Abdisalan M. Noor

**Affiliations:** 1 Kenya Medical Research Institute-Wellcome Trust Research Programme, Nairobi, Kenya; 2 National Malaria Control Programme, Ministry of Health, Nairobi, Kenya; 3 Division of Monitoring and Evaluation, Health Research Development and Health Informatics, Ministry of Health, Nairobi, Kenya; 4 E&K Consulting Firm, Nairobi, Kenya; 5 Centre for Tropical Medicine and Global Health, Nuffield Department of Clinical Medicine, University of Oxford, Oxford, UK; 6 Malaria Branch, Division of Parasitic Diseases and Malaria, Center for Global Health, U.S. Centers for Disease Control and Prevention, Nairobi, Kenya; 7 U.S. President’s Malaria Initiative - Kenya, Nairobi, Kenya

## Abstract

**Background:**

Health facility-based data reported through routine health information systems form the primary data source for programmatic monitoring and evaluation in most developing countries. The adoption of District Health Information Software (DHIS2) has contributed to improved availability of routine health facility-based data in many low-income countries. An assessment of malaria indicators data reported by health facilities in Kenya during the first 5 years of implementation of DHIS2, from January 2011 to December 2015, was conducted.

**Methods:**

Data on 19 malaria indicators reported monthly by health facilities were extracted from the online Kenya DHIS2 database. Completeness of reporting was analysed for each of the 19 malaria indicators and expressed as the percentage of data values actually reported over the expected number; all health facilities were expected to report data for each indicator for all 12 months in a year.

**Results:**

Malaria indicators data were analysed for 6235 public and 3143 private health facilities. Between 2011 and 2015, completeness of reporting in the public sector increased significantly for confirmed malaria cases across all age categories (26.5–41.9%, p < 0.0001, in children aged <5 years; 30.6–51.4%, p < 0.0001, in persons aged ≥5 years). Completeness of reporting of new antenatal care (ANC) clients increased from 53.7 to 70.5%, p < 0.0001). Completeness of reporting of intermittent preventive treatment in pregnancy (IPTp) decreased from 64.8 to 53.7%, p < 0.0001 for dose 1 and from 64.6 to 53.4%, p < 0.0001 for dose 2. Data on malaria tests performed and test results were not available in DHIS2 from 2011 to 2014. In 2015, sparse data on microscopy (11.5% for children aged <5 years; 11.8% for persons aged ≥5 years) and malaria rapid diagnostic tests (RDTs) (8.1% for all ages) were reported. In the private sector, completeness of reporting increased significantly for confirmed malaria cases across all age categories (16.7–23.1%, p < 0.0001, in children aged <5 years; 19.4–28.6%, p < 0.0001, in persons aged ≥5 years). Completeness of reporting also improved for new ANC clients (16.2–23.6%, p < 0.0001), and for IPTp doses 1 and 2 (16.6–20.2%, p < 0.0001 and 15.5–20.5%, p < 0.0001, respectively). In 2015, less than 3% of data values for malaria tests performed were reported in DHIS2 from the private sector.

**Conclusions:**

There have been sustained improvements in the completeness of data reported for most key malaria indicators since the adoption of DHIS2 in Kenya in 2011. However, major data gaps were identified for the malaria-test indicator and overall low reporting across all indicators from private health facilities. A package of proven DHIS2 implementation interventions and performance-based incentives should be considered to improve private-sector data reporting.

**Electronic supplementary material:**

The online version of this article (doi:10.1186/s12936-017-1973-y) contains supplementary material, which is available to authorized users.

## Background

Reliable health data are essential for health planners and programme implementers to make informed decisions about resource allocations to prevent morbidity and mortality [1]. Historically, population-based health surveys have been conducted periodically to collect retrospective data to inform planning and policy needs [2]. However, nationally representative health surveys are expensive, infrequent and might not reflect the current situation, particularly in a dynamic health environment. By contrast, health facility-based data are collected on a routine basis and have the potential to present more real-time data for decision making, allowing disease control programmes to direct interventions in a more timely manner [2]. Health facility-based data reported through routine health information systems form the primary data source for national health planning, health surveillance and programmatic monitoring and evaluation in most developing countries [3, 4]. Escalation of malaria control activities in Africa has led to increased demand for more robust data to track changes in transmission patterns and to develop interventions targeted to local heterogeneous conditions [5]. Routine data from health systems is critical for the design and implementation of malaria control programmes so that resources can be targeted to the populations and areas that are most in need [6]. However, the quality of routine data has often been poor with a general lack of timeliness, completeness and accuracy [7–11]. The lack of quality data has resulted in challenges in using routine data to monitor and evaluate health interventions [12–14]. The World Health Organization (WHO) relies on modelling to estimate malaria morbidity and mortality trends in most countries in the African region due to incomplete routine data [15, 16]. However, the situation is now changing in many countries due to increased recognition of the importance of robust routine data to accurately track health progress and rapid developments in information technology [5, 17].

The adoption of innovative, free and open source web-based reporting software such as the District Health Information Software 2 (DHIS2) has contributed to improved availability of routine health facility-based data in many developing countries [4, 18–21]. Following successful customization, testing and piloting, Kenya adopted DHIS2 as the routine health data reporting platform in 2011 [22]. The current online DHIS2 database is structured by administrative units with individual health facilities assigned to 299 sub-counties and 47 counties. Health facilities report monthly data on diseases, commodities and service delivery through DHIS2. Data are collected through a paper-based system of registers, tally sheets, and monthly data reporting forms at each health facility. Due to poor internet connectivity and lack of infrastructure in most rural health facilities, the collated monthly data are sent to the sub-county level where they are entered into the web-based DHIS2 [21]. The software captures and remotely stores facility-level data by month and can generate quarterly or yearly reports by different administrative levels. The data entered and stored into the system are available to registered users and other stakeholders through a password protected log-in system. Reporting rates for outpatient data have increased substantially since the introduction of DHIS2, and the system has high acceptance and utilization by stakeholders [21–23].

In Kenya, malaria accounts for approximately 18% of outpatient visits at health facilities [24]. Clinical and confirmed malaria cases encountered in outpatient clinics are reported via DHIS2. The DHIS2 data repository contains substantial numbers of malaria cases reported across all health facilities in Kenya. However, routine surveillance reports published by the National Malaria Control Programme (NMCP) have consistently shown anomalies in data reported via DHIS2 [25–31]. A nationally-representative, health facility-based survey that tested outpatients with suspected malaria in October and November 2014 found no confirmed malaria cases at 47% of the surveyed facilities [32]. However, confirmed malaria cases were reported in routine DHIS2 at the same facilities during the same time period [32]. In order to fully utilize routine malaria surveillance data for policy and programming decisions, the NMCP and stakeholders must have confidence in the data. The quality of data reported via DHIS2 during the first 5 years of implementation, from January 2011 to December 2015, was evaluated through an analysis of completeness of 19 malaria indicators reported monthly by health facilities.

## Methods

Nineteen malaria data indicators reported monthly by health facilities were extracted from the DHIS2 online database [33] in February 2016. These included total number of clinical and confirmed malaria cases, artemether–lumefantrine (AL) treatments, intermittent preventive treatment in pregnancy (IPTp) with sulfadoxine–pyrimethamine doses, long-lasting insecticidal bed nets (LLINs) distributed via antenatal care (ANC) and children health clinics and malaria tests performed (Table 1). Monthly data were extracted separately for each sub-county, disaggregated by health facility and arranged chronologically. The assembled sub-county data sets were merged into one database for the country.

**Table 1:** List of malaria indicators assessed in the Kenya District Health Information Software 2

Data indicators
Number of malaria cases
Number of clinical malaria cases in children <5 years
Number of clinical malaria cases in persons ≥5 years
Number of confirmed malaria cases in children <5 years
Number of confirmed malaria cases in persons ≥5 years
Artemether–lumefantrine treatments
Number of patients treated with AL weight band 5–14 kg
Number of patients treated with AL weight band 15–24 kg
Number of patients treated with AL weight band 25–34 kg
Number of patients treated with AL weight band ≥35 kg
Antenatal care and IPTp^a^
Number of new ANC clients
Number of ANC clients who received first dose of IPTp
Number of ANC clients who received second dose of IPTp
Long-lasting insecticidal bed nets^b^
Number of LLINs distributed at ANC clinics
Number of LLINs distributed at child health clinics
Diagnostic test indicators
Number of malaria blood slides examined in children <5 years
Number of malaria blood slides examined in persons ≥5 years
Number of positive malaria blood slides in children <5 years
Number of positive malaria blood slides in persons ≥5 years
Number of malaria RDTs examined^c^
Number of positive malaria RDTs^c^

*ANC* antenatal care, *IPTp* intermittent preventive treatment in pregnancy with sulfadoxine–pyrimethamine, *RDT* rapid diagnostic test

^a^Limited to 14 counties where malaria transmission is moderate-to-high

^b^Limited to 36 counties where nets are routinely distributed

^c^RDT indicators were not reported by age category

Completeness of indicator data reporting was expressed as the percentage of data values actually reported over the expected number given that all health facilities were expected to report each indicator for all 12 months in a year. Completeness of reporting was calculated by summing up the number of months with a data value reported for a given indicator across all health facilities in a year and dividing by the expected number of values (i.e., 12 months × total number of health facilities). All blank data fields were considered to be unreported data. Analysis was performed separately for public and private facilities and by epidemiological zones representing malaria transmission risk (i.e., the endemic zones of stable malaria around Lake Victoria and coast regions; the seasonal-transmission zone in the arid and semi-arid areas of northern and south-eastern regions; the highland epidemic-prone zone of the western region; and low-risk zone in the central highlands including Nairobi) [23].

Analysis of completeness of reporting of IPTp doses given to pregnant women was restricted to 1490 public and 709 private health facilities located in 14 counties in the lake-and coast-endemic zones. The analysis was restricted because the intervention is targeted only to HIV-negative women living in areas of moderate-to-high malaria transmission [23, 34]. Similarly, analysis of completeness of reporting of LLINs distributed was restricted to 4793 public and 2336 private health facilities located in 36 counties targeted for routine distribution of nets through ANC and child health clinics. Analysis of completeness of malaria testing indicators was only reported for 2015 because data for previous years were not available. The change in indicator data reporting completeness over time is presented as a percentage-point increase or decrease in data values reported in 2015 compared to 2011 except for artemether–lumefantrine (AL) treatment indicators for which 2012 was used as the baseline year due to limited data reported in 2011. The percentage of health facilities in the public sector that reported data for each indicator for all 12 months in a year was also determined. All data were cleaned and analysed in Excel 2013 (Microsoft Corporation, Seattle, WA) and Stata version 12 (StataCorp, College Station, TX).

## Results

### Description of health facilities analysed

Data were extracted from all 10,090 health facilities registered in the DHIS2 online database at the time of the study. During data cleaning, a total of 712 health facilities were excluded from analysis; 544 were health facilities that offered specialized services, 93 were duplicated facilities due to multiple spellings for the same health facility, 39 were unclassified facilities (i.e., facility type and managing authority not specified), and 36 were reporting units representing aggregated data from several facilities already included. Data were therefore analysed for 9378 (92.9%) of the health facilities registered in DHIS2 at the time of the study. Of these facilities, 6235 (66.5%) were public and 3143 (33.5%) were private (Table 2).

**Table 2:** Description of health facilities by type and sector

Facility type (N = 9378)	Public		Private	
	n	%	n	%
Dispensaries and clinics	4776	76.6	2932	93.3
Health centres	1032	16.6	61	1.9
Hospitals	427	6.8	150	4.8
Total	6235	66.5	3143	33.5

### Completeness of data reported in public health facilities

Within the public sector, the percentage of data values reported for confirmed malaria cases increased in children aged <5 years (26.5–41.9%, p < 0.0001) and in persons aged ≥5 years (30.6–51.4%, p < 0.0001) (Table 3). Conversely, the percentage of data values reported for clinical (i.e., non-confirmed) malaria cases decreased in children aged <5 years (55.6–27.0%, p < 0.0001) and in persons aged ≥5 years (56.4–30.3%, p < 0.0001). There were small but significant declines in the percentage of data values reported for patients treated with the different AL treatment weight bands (declines ranged from 3.0% for AL ≥35 kg (adult dosing) to 7.6% for AL 15–24 kg (pediatric dosing). The percentage of data values reported for new ANC clients increased (53.6–70.3%, p < 0.0001) while data values reported for IPTp doses in the 14 targeted counties decreased by 11%, p < 0.0001. In the 36 counties targeted for routine distribution of LLINs, the percentage of data values reported for LLINs distributed via ANC clinics increased from 47.1 to 61.6% (p < 0.0001) and via child health clinics from 20.3 to 41.5% (p < 0.0001). In 2015, sparse malaria testing data were reported for microscopy (11.5% for children aged <5 years; 11.8% for persons aged ≥5 years) and RDT (8.1% for all ages). Completeness of reporting based on the percentage of public health facilities that reported data on each indicator for all 12 months each year was much lower overall although the trends were similar to those presented in Table 3 (Additional file 1).

**Table 3:** Percentage of malaria indicator data values reported by public health facilities through the District Health Information Software 2 in Kenya, 2011–2015

Malaria indicators	Percentage of data values reported by year					% point change	*p* value
	2011	2012	2013	2014	2015		
Malaria cases							
Confirmed malaria (<5 years)	26.5	30.2	37.9	38.5	41.9	15.4	0.0001
Confirmed malaria (≥5 years)	30.6	35.9	45.5	46.8	51.4	20.8	0.0001
Clinical malaria (<5 years)	55.6	58.9	46.5	42.5	27.0	−28.6	0.0001
Clinical malaria (≥5 years)	56.4	60.9	49.4	45.6	30.3	−26.0	0.0001
Artemether–lumefantrine treatments							
AL weight band 5–14 kg		37.3	35.9	30.7	30.8	−6.5	0.0001
AL weight band 15–24 kg		35.2	33.5	30.0	27.6	−7.6	0.0001
AL weight band 25–34 kg		32.7	29.3	26.1	28.2	−4.5	0.0001
AL weight band ≥35 kg		37.6	37.5	32.0	34.6	−3.0	0.0001
Antenatal care and IPTp							
New ANC clients	53.7	61.5	64.8	67.7	70.5	16.8	0.0001
IPTp dose 1^a^	64.8	69.5	72.2	72.9	53.7	−11.1	0.0001
IPTp dose 2^a^	64.6	69.2	72.2	72.5	53.3	−11.3	0.0001
Long-lasting insecticidal bed nets^b^							
LLINs via ANC clinics	47.1	45.0	55.7	57.3	61.6	14.5	0.0001
LLINs via child health clinics	20.3	38.5	46.7	42.8	41.5	21.2	0.0001
Diagnostic test indicators^c^							
Blood slide tested (<5 years)	0.0	0.0	0.0	0.1	11.5		
Blood slide positive (<5 years)	0.0	0.0	0.0	0.1	8.5		
Blood slide tested (≥5 years)	0.0	0.0	0.0	0.1	12		
Blood slide positive (≥5 years)	0.0	0.0	0.0	0.1	9.4		
RDT tested^d^	0.0	0.0	0.0	0.0	8.1		
RDT positive^d^	0.0	0.0	0.0	0.0	5.4		

*AL* artemether–lumefantrine, *ANC* antenatal care, *IPTp* intermittent preventive treatment in pregnancy with sulfadoxine–pyrimethamine, *LLINs* long-lasting insecticidal bed nets, *RDT* rapid diagnostic test

^a^Analysis was restricted to 1490 health facilities in the lake-and coast-endemic zones where the IPTp intervention was targeted

^b^Analysis was restricted to 4793 health facilities in 36 counties targeted for routine distribution of LLINs

^c^Data only available for 2015

^d^RDT data not reported by age category

**Fig. 1:**
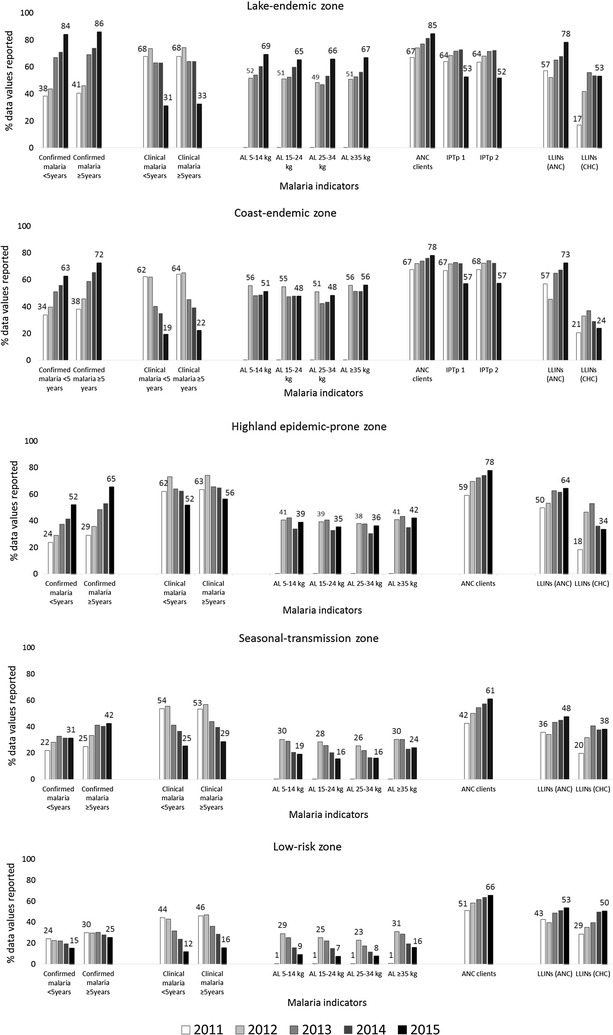
Percentage of malaria data values submitted by public health facilities to District Health Information Software 2 in Kenya by transmission zones, 2011–2015. *AL* artemether–lumefantrine, *ANC* antenatal care, *CHC* child health clinic, *IPTp* intermittent preventive treatment in pregnancy with sulfadoxine–pyrimethamine (IPTp intervention is only targeted in the lake-and coast-endemic zones), *LLINs* long-lasting insecticidal bed nets

Analysis by malaria-risk zones showed significant increases in the percentage of data values reported for confirmed malaria cases in all except the low-risk zone (Fig. 1). The increase was highest in the lake-endemic zone (38–84% in children aged <5 years, p < 0.0001 and 41–86% in persons aged ≥5 years, p < 0.0001). The percentage of data values reported for clinical malaria cases declined significantly across all zones. The decline was greatest in the coast-endemic zone (62–19%, p < 0.0001 in children aged <5 years; 64–22%, p < 0.0001 in persons aged ≥5 years) and lowest in the highland epidemic-prone zone (62–52%, p < 0.0001 in children aged <5 years; 63–56%, p < 0.0001 in persons aged ≥5 years). Concurrent with a decrease in reporting of clinical malaria cases, the percentage of data values reported for the four AL treatment weight bands declined in all except the lake-endemic zone. The decreases were greatest in the low-risk zone (ranging from 15 to 20% decreases, all p < 0.0001) and smallest in the highland epidemic-prone zone (ranging from 2 to 4% decreases, all p < 0.0001) for all treatment weight bands. The percentage of data values reported for new ANC clients increased in all zones from 2011 to 2015 (range; 11 to 19%, p < 0.0001 for all). Reporting of LLINs distributed through ANC and child health clinics increased across all zones. The largest increase was in the lake-endemic zone with 21 and 36% increase for LLINs distributed via ANC and child health clinics, respectively (p < 0.0001 for both).

### Completeness of data reported in private health facilities

**Fig. 2:**
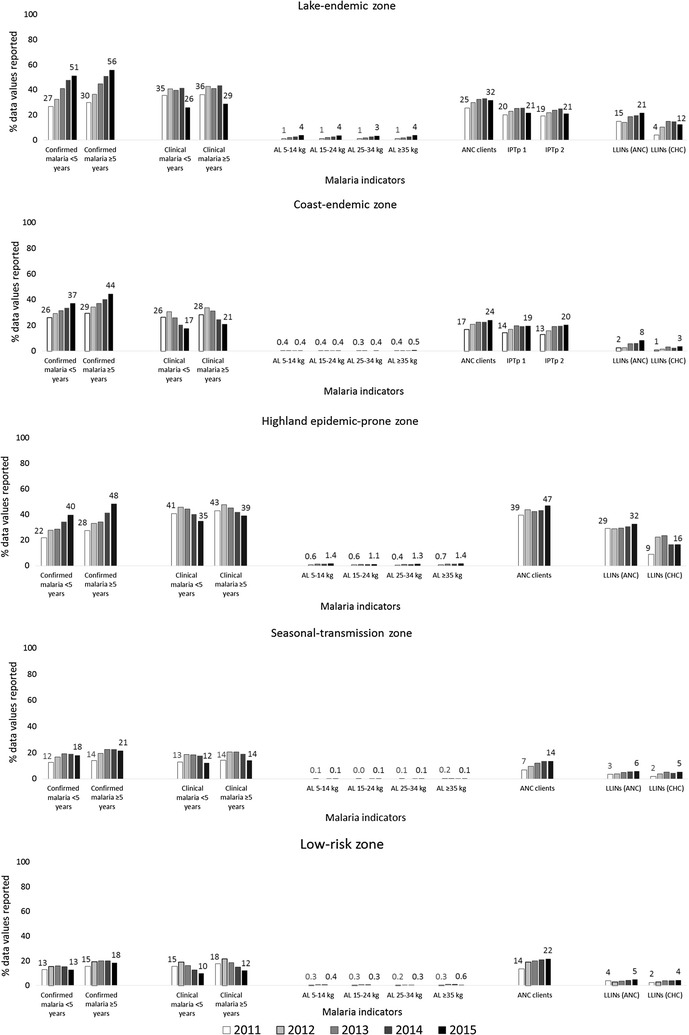
Percentage of malaria data values submitted by private health facilities to District Health Information Software 2 in Kenya by transmission zones, 2011–2015. *AL* artemether–lumefantrine, *ANC* antenatal care, *CHC* child health clinic, *IPTp* intermittent preventive treatment in pregnancy with sulfadoxine–pyrimethamine (IPTp intervention is only targeted in the lake-and coast-endemic zones), *LLINs* long-lasting insecticidal bed nets

Overall, analysis of data reporting completeness from private health facilities showed trends that were similar to those seen in the public sector. However, the completeness of reporting in 2015 compared to 2011 was much lower for all indicators in private health facilities compared to public health facilities (Table 4; Fig. 2). A significant increase was observed in the percentage of data values reported for confirmed malaria cases across all ages (16.7–23.1%, p < 0.0001, in children aged <5 years; 19.4–28.6%, p < 0.0001, in persons aged ≥5 years). Other significant increases were observed in data values reported for new ANC clients (16.2–23.6%, p < 0.0001) and LLINs distributed via ANC (7.9–11.0%, p < 0.0001) and child health clinics (3.0–6.8%, p < 0.0001) (Table 4). Less than 1% of data values for AL treatment weight bands were reported by private health facilities for any given year. Less than 3% of data values for malaria testing indicators were reported by private health facilities in 2015. Analysis by malaria-risk zones showed that the largest increase in confirmed malaria cases reporting was in the lake-endemic zone (27–51%, p < 0.0001, in children aged <5 years; 30–56%, p < 0.0001, in persons aged ≥5 years). Clinical malaria reporting declined in all zones (p < 0.0001 for all). Reporting of new ANC clients increased by 7–8% across all zones (p < 0.0001 for all) (Fig. 2).

**Table 4:** Percentage of malaria data values reported by private health facilities through the District Health Information Software 2 in Kenya, 2011–2015

Malaria indicators	Percentage of data values reported by year					% point change	*p* value
	2011	2012	2013	2014	2015		
Malaria cases							
Confirmed malaria (<5 years)	16.7	20.3	20.2	23.2	23.1	6.4	0.0001
Confirmed malaria (≥5 years)	19.4	24.0	24.1	28.0	28.6	9.2	0.0001
Clinical malaria (<5 years)	20.6	25.0	20.5	19.8	15.0	−5.6	0.0001
Clinical malaria (≥5 years)	22.4	27.3	22.7	22.1	17.7	−4.7	0.0001
Artemether–lumefantrine treatments							
AL weight band 5–14 kg		0.4	0.7	0.8	0.8	0.4	0.0001
AL weight band 15–24 kg		0.4	0.7	0.8	0.7	0.3	0.0001
AL weight band 25–34 kg		0.3	0.5	0.7	0.7	0.4	0.0001
AL weight band ≥35 kg		0.4	0.8	1.0	0.9	0.5	0.0001
Antenatal care and IPTp							
New ANC clients	16.2	20.6	20.1	22.9	23.6	7.5	0.0001
IPTp1^a^	16.6	19.5	22.0	22.1	20.2	3.6	0.0001
IPTp2^a^	15.5	18.4	21.2	22.0	20.5	5.0	0.0001
Long-lasting insecticidal bed nets^b^							
LLINs via ANC clinics	7.9	7.6	9.2	9.9	11.0	3.1	0.0001
LLINs via child health clinics	3.0	6.2	7.8	6.8	6.8	3.8	0.0001
Diagnostic test indicators^c^							
Blood slide tested (<5 years)	0.0	0.0	0.0	0.0	2.9		
Blood slide positive (<5 years)	0.0	0.0	0.0	0.0	2.1		
Blood slide tested (≥5 years)	0.0	0.0	0.0	0.0	3.5		
Blood slide positive (≥5 years)	0.0	0.0	0.0	0.0	2.9		
RDT tested^d^	0.0	0.0	0.0	0.0	1.0		
RDT positive^d^	0.0	0.0	0.0	0.0	0.7		

*AL* artemether–lumefantrine, *ANC* antenatal care, *IPTp* intermittent preventive treatment in pregnancy with sulfadoxine–pyrimethamine, *LLINs* long-lasting insecticidal bed nets, *RDT* rapid diagnostic test

^a^Analysis was restricted to 709 private health facilities in the lake-and coast-endemic zones where the IPTp intervention was targeted

^b^Analysis was restricted to 2366 private health facilities in 36 counties targeted for routine distribution of LLINs

^c^Data only available for 2015

^d^RDT data not reported by age category

## Discussion

Routine malaria data for 19 indicators reported via the Kenya DHIS2 were assessed for completeness from 2011 to 2015. The assessment showed improvements in both national and sub-national reporting completeness for malaria indicator data over the first 5 years of DHIS2 implementation in Kenya. Completeness of data reporting in the public sector was much higher than in the private sector. Improvements in reporting completeness in the public sector may be attributed to substantial infrastructure and human resource investments for DHIS2 implementation including longitudinal training and support [22]. In addition, the public sector has higher demands for data to meet the reporting requirements of external donors and forecast commodity needs. For example, DHIS2 data fulfils the Global Fund to Fight AIDS, Tuberculosis and Malaria grant reporting requirements and informs forecasting for commodity procurements including RDTs, AL and LLINs [35]. The higher demand for and use of public sector health data has also led to additional disease-specific investments to improve reporting.

Private-sector facilities, in contrast, do not rely on donor-funded commodities, and the demand for data from external users is low. Kenya, however, has a large and robust private health sector comprising half of all health facilities [36]. In 2015, 25% of children who had a fever in the 2 weeks preceding a large household malaria survey sought treatment from private health facilities [37]. The private sector would be a rich source of malaria data and increase the generalizability of national and sub-national trends if reported. The Kenya Malaria Strategy recognizes the contribution of the private sector in providing care for malaria patients [23]. The NMCP provides support to the private sector primarily through the Affordable Medicines Facility-malaria to increase the affordability and availability of quality-assured AL via subsidies but also through malaria case management trainings [23]. However, data reporting from private health facilities remains poor because there is little incentive to report and no enforcement of the data reporting policy [38]. Providing a DHIS2 implementation package to private sector health facilities that includes training, standardized reporting tools and software support might have a similar positive impact as realized in the public sector. Performance-based incentives might also be considered; private health facilities are likely to be motivated by facility-based incentive programmes that give them an opportunity to earn additional resources [39].

A major limitation of the malaria indicator data was the inability to distinguish true missing values (i.e., no data reported) from ‘*zero*’ values (i.e., no events captured) because both scenarios appeared as blank data entries in DHIS2. Health facilities in Kenya do not usually report ‘*zero*’ values when no events are captured. All blank data entries were, therefore, classified as missing (i.e., no data reported). As a result, the completeness of reporting observed likely under-represented the true situation. For example, in low-risk and seasonal-transmission zones, the majority of health facilities did not report confirmed malaria cases. Based on the very low prevalence of malaria in these zones, it is probable that the majority of health facilities diagnosed zero confirmed malaria cases for at least some months of the year, which is consistent with findings from a recent health-facility survey [32]. Currently in DHIS2, facilities with no confirmed malaria cases that do not report a ‘*zero*’ value and facilities that diagnose malaria cases but do not report both show blank fields. DHIS2 should require data fields for key malaria indicators to be filled in using conditional rules requiring input of an integer value including ‘*zero*’ and unfilled fields defaulting to ‘*NDR*’ (i.e., no data reported) or another coded value prior to data submission.

The relationship between program implementation and reporting is also critical to the interpretation of surveillance data and related to zero-reporting. Without zero-reporting, it is impossible to determine from malaria-indicator data alone if a program service or commodity is not delivered, not reported or both. For example, despite over 90% of public sector health facilities having diagnostic capacity and approximately 40% reporting confirmed malaria cases in 2013, none reported diagnostic testing data via DHIS2 in 2013 [40]. This is an example of service delivery but no reporting via DHIS2. Although completeness of reporting does not necessarily reflect programme implementation, facilities have incentive to report when the data drives actions such as malaria commodity procurements and deliveries.

Analysis by malaria-risk zones showed that the lake-endemic zone had the greatest reporting completeness improvements for confirmed malaria cases, AL treatments, and LLINs distributed via ANC and child health clinics. Because the majority of malaria cases are in the lake-endemic zone, the NMCP and key partners have focused malaria prevention and control interventions in the eight counties located in this zone since 2013 [41]. Improvements in malaria indicator data reporting completeness are likely a result of substantial malaria and other disease programming investments, including strengthening supply chain management for commodities and surveillance, in the zone [41].

Overall, there were significant increases in completeness of reporting of confirmed malaria cases, new ANC clients, and LLIN distribution via ANC and child health clinics in both the public and private sectors while data values reported for clinical malaria cases declined. The increase in reporting completeness of confirmed malaria cases was probably related to the national expansion of malaria RDTs. In late 2012, the NMCP implemented policy to distribute malaria RDTs to all public sector health facilities in Kenya. *CareStart* Malaria histidine-rich protein 2 (HRP2) *Plasmodium falciparum* RDTs were supplied to all health facilities through the Kenya Medical Supplies Agency [25]. Consequently, the percentage of public sector health facilities with functional capacity to diagnose malaria increased from 59% in 2011 to 97% by the end of 2015 [40]. However, despite significant improvements, the assessment found that the highland epidemic-prone zone continued to report a relatively high percentage of data values for clinical malaria cases. The inability of health workers to establish differential diagnosis for persons with malaria-test negative results might explain continued reporting of clinical malaria cases despite availability of malaria diagnostics [35]. Sustained availability of malaria diagnostics in all health facilities, parasitological diagnosis of malaria for all persons before treatment and capacity to appropriately manage malaria test-negative patients are all critical components required to achieve the national target of 100% parasitological diagnosis of all persons with suspected malaria presenting to a health provider in Kenya [23].

Although 90% of pregnant women make at least one ANC visit, only 70% of public sector facilities reported new ANC visit [37, 42]. The indicator, attendance at first ANC visit, is more frequently reported compared to other malaria indicators due, in part, to its widespread use as a tracer indicator in the WHO data quality assessment framework [4, 43]. Attendance at first ANC visit, a national indicator, and IPTp 1 and IPTp 2, sub-national indicators, both showed increasing trends for reporting completeness from 2011 to 2014. However, in 2015, the percentage of public-sector facilities reporting IPTp 1 and 2 declined by 19 percentage points compared to 2014 despite the continued positive trend in attendance at first ANC visit reporting. It seems unlikely that facilities would continue reporting attendance at first ANC visit but stop reporting IPTp 1 and 2 doses. Kenya experienced country-wide stock-outs of sulfadoxine–pyrimethamine in 2015, which were reported in national malaria surveillance bulletins and biennial quality-of-care survey [41]. Thus, the decline in reporting completeness of IPTp 1 and 2 doses seems more likely a reflection of interrupted service delivery due to lack of sulfadoxine–pyrimethamine at the facility-level in 2015; data interpretation would be more direct if facilities had reported ‘*zeros*’ for IPTp doses.

The overall gains in reporting completeness of malaria data indicators in Kenya could be attributed, in part, to technical expertise from the Ministry of Health and strong partnerships with development, technical and implementing partners, who have contributed financial, technical and operational support [23]. Similar expertise and partnerships are being developed at the county level, which have advanced rapidly since 2013. The strong national malaria strategic plan framework has led to robust monitoring and evaluation of malaria indicators such as quarterly national malaria surveillance bulletins since 2012, biennial quality-of-care surveys since 2010, and regular national household surveys [23]. For example, quarterly national malaria surveillance bulletins highlight counties with reporting rates below 60% [25–31, 44–46]. Regular data reviews and technical working groups at both the national and county levels monitor the progress of key malaria indicators and support is provided to counties to increase reporting rates to >80% to ensure that the data reported nationally is both generalizable and representative.

There were important gaps in the reporting system as a result of programmatic limitations, which were reflected in the analysis. The lack of data for malaria tests performed and test-positive results was a major gap identified in DHIS2. Prior to the national introduction of malaria RDTs, malaria testing was predominantly performed by microscopy and reported on a quarterly basis through a parallel laboratory management information system. Following the national introduction of malaria RDTs, new data forms to capture persons with suspected malaria tested by RDTs and microscopy and results by type of test were developed and integrated into DHIS2 in 2014. However, the transition to reporting malaria-test data through DHIS2 has been slow as shown by the paucity of data reported in 2015.

The overarching goal of the Kenya national malaria strategy is elimination [23, 47]. Zero-reporting is a key surveillance strategy successfully used to increase sensitivity in the polio eradication programme, which was later extended to other disease elimination and eradication programmes [48]. Kenya should consider adopting a zero-reporting policy and instituting DHIS2 system-based solutions along with revised training and support modules to improve surveillance. High reporting completeness for malaria indicators, including zero-reporting, could provide sufficient data to determine when sub-national regions and the country meet the required WHO threshold (i.e., slide positivity rate of <5% during the peak malaria season) to move to the malaria pre-elimination phase [49]. Finally, due to the very large number of observations included in the analysis (i.e., all health facilities), very small changes over time, which were statistically significant, may not necessarily indicate sustained systematic improvement. Broader contextual changes were therefore considered in the interpretation of the results.

## Conclusions

There have been sustained improvements in the completeness of data reporting for most key malaria indicators since the adoption of DHIS2 in Kenya in 2011. However, major gaps were identified including the lack of data reported for malaria tests performed, no zero-reporting and overall low reporting from private health facilities. Ongoing efforts to integrate malaria-test data into DHIS2 should improve indicator availability and help determine Kenya’s progress towards meeting national and international targets. A package of proven DHIS2 implementation interventions and performance-based incentives should be considered to improve private-sector data reporting.

## Additional file

**Additional file 1.** Percentage of public health facilities that reported malaria indicator data values to the District Health Information Software 2 for all 12 months each year in Kenya, 2011–2015.

## References

[1] WHO (2008). Toolkit on monitoring health systems strengthening.

[2] WHO. Assessment of health facility data quality; data quality report card Uganda, 2010–2011. Geneva: World Health Organization; 2011.

[3] Gimbel S, Micek M, Lambdin B, Lara J, Karagianis M, Cuembelo F (2011). An assessment of routine primary care health information system data quality in Sofala Province, Mozambique. Popul Health Metr.

[4] Nisingizwe MP, Iyer HS, Gashayija M, Hirschhorn LR, Amoroso C, Wilson R (2014). Toward utilization of data for program management and evaluation: quality assessment of five years of health management information system data in Rwanda. Glob Health Action.

[5] Gething PW, Battle KE, Bhatt S, Smith DL, Eisele TP, Cibulskis RE (2014). Declining malaria in Africa: improving the measurement of progress. Malar J.

[6] WHO (2012). Disease surveillance for malaria control.

[7] Mavimbe JC, Braa J, Bjune G (2005). Assessing immunization data quality from routine reports in Mozambique. BMC Public Health.

[8] Hahn D, Wanjala P, Marx M (2013). Where is information quality lost at clinical level? A mixed-method study on information systems and data quality in three urban Kenyan ANC clinics. Global Health Action.

[9] Maokola W, Willey B, Shirima K, Chemba M, Armstrong Schellenberg J, Mshinda H (2011). Enhancing the routine health information system in rural southern Tanzania: successes, challenges and lessons learned. Trop Med Int Health.

[10] Nyamtema AS (2010). Bridging the gaps in the health management information system in the context of a changing health sector. BMC Med Inform Decis Mak.

[11] Kihuba E, Gathara D, Mwinga S, Mulaku M, Kosgei R, Mogoa W (2014). Assessing the ability of health information systems in hospitals to support evidence-informed decisions in Kenya. Global health action.

[12] Rowe AK, Kachur SP, Yoon SS, Lynch M, Slutsker L, Steketee RW (2009). Caution is required when using health facility-based data to evaluate the health impact of malaria control efforts in Africa. Malar J.

[13] Mate KS, Bennett B, Mphatswe W, Barker P, Rollins N (2009). Challenges for routine health system data management in a large public programme to prevent mother-to-child HIV transmission in South Africa. PLoS ONE.

[14] Amouzou A, Kachaka W, Banda B, Chimzimu M, Hill K, Bryce J (2013). Monitoring child survival in ‘real time’using routine health facility records: results from Malawi. Trop Med Int Health.

[15] WHO (2014). World malaria report 2014.

[16] World Health Organization (2015). World malaria report 2015.

[17] Chan M, Kazatchkine M, Lob-Levyt J, Obaid T, Schweizer J, Sidibe M (2010). Meeting the demand for results and accountability: a call for action on health data from eight global health agencies. PLoS Med.

[18] WHO (2014). U.S. Agency for International Development and University of Oslo. Health facility and community data toolkit.

[19] Kiberu VM, Matovu JK, Makumbi F, Kyozira C, Mukooyo E, Wanyenze RK (2014). Strengthening district-based health reporting through the district health management information software system: the Ugandan experience. BMC Med Inform Decis Mak.

[20] Mphatswe W, Mate K, Bennett B, Ngidi H, Reddy J, Barker P (2012). Improving public health information: a data quality intervention in KwaZulu-Natal, South Africa. Bull World Health Organ.

[21] Karuri J, Waiganjo P, Daniel O, Manya A (2014). DHIS2: the tool to improve health data demand and use in Kenya. J Health Inform Dev Countries.

[22] Manya A, Braa J, Øverland LH, Titlestad OH, Mumo J, Nzioka C. National roll out of District Health Information Software (DHIS 2) in Kenya, 2011—Central server and Cloud based infrastructure. IST-Africa 2012 Conference Proceedings 2012.

[23] MOH (2014). The Kenya malaria strategy 2009–2018 (Revised 2014).

[24] MOH (2015). Kenya annual malaria report 2013–2014.

[25] NMCP (2012). Malaria surveillance bulletin Issue No. 3.

[26] NMCP (2013). Malaria surveillance bulletin Issue No. 4.

[27] NMCP (2013). Malaria surveillance bulletin Issue No. 5.

[28] NMCP (2013). Malaria surveillance bulletin Issue No. 7.

[29] NMCP (2014). Malaria surveillance bulletin Issue No. 10.

[30] NMCP (2014). Malaria surveillance bulletin Issue No. 11.

[31] NMCP (2015). Malaria Surveillance Bulletin Issue No. 13.

[32] Githinji S, Noor AM, Malinga J, Macharia PM, Kiptui R, Omar A (2016). A national health facility survey of malaria infection among febrile patients in Kenya, 2014. Malar J.

[33] Kenya Health Information System. http://www.hiskenya.org. Accessed on 29 Feb 2016.

[34] WHO (2013). Policy brief for the implementation of intermittent preventive treatment of malaria in pregnancy using sulfadoxine-pyrimethamine (IPTp-SP).

[35] U.S. President’s Malaria Initiative (2015). Kenya Malaria Operational Plan FY 2015.

[36] MOH (2013). Kenya service availability and readiness assessment mapping (SARAM) report 2013.

[37] National Malaria Control Programme (NMCP) Kenya National Bureau of Statistics, ICF International (2016). Kenya malaria indicator survey 2015.

[38] Ministry of Medical Services and Ministry of Public Health and Sanitation (2012). Health information system policy 2010–2030.

[39] Menya D, Platt A, Manji I, Sang E, Wafula R, Ren J (2015). Using pay for performance incentives (P4P) to improve management of suspected malaria fevers in rural Kenya: a cluster randomized controlled trial. BMC Med.

[40] Machini B, Nyandigisi A, Kigen S, Memusi D, Kimbui R, Malinga J (2016). Monitoring outpatient malaria case management under the 2010 diagnostic and treatment policy in Kenya-Progress 2010–2015.

[41] U.S. President’s Malaria Initiative (2016). Kenya Malaria Operational Plan FY 2016.

[42] KNBS (2015). Kenya demographic and health survey 2014.

[43] WHO. Guide to the health facility data quality report card. Geneva: World Health Organization; 2014. http://www.who.int/healthinfo/topics_standards_tools_data_quality_analysis/en/index.html. Accessed 10 Jan 2017.

[44] NMCP (2014). Malaria surveillance bulletin Issue No. 12.

[45] NMCP (2015). Malaria surveillance bulletin Issue No. 14.

[46] NMCP (2015). Malaria surveillance bulletin Issue No. 15.

[47] MOH. Transforming Health—Accelerating Attainment of Universal Health Coverage; Kenya Heath Sector Strategic and investment Plan (KHSSP), 2014–2018.

[48] Tangermann RH, Lamoureux C, Tallis G, Goel A (2017). The critical role of acute flaccid paralysis surveillance in the Global Polio Eradication Initiative. Int Health.

[49] WHO (2012). Disease surveillance for malaria elimination: an operational manual.

