# Supplementary material for: Completeness of malaria indicator data reporting via the District Health Information Software 2 in Kenya, 2011–2015
Source: Malar J. 2017 Aug 17;16:344. doi: 10.1186/s12936-017-1973-y (PMC5561621; doi:10.1186/s12936-017-1973-y)
Supplement: Supplementary file 1 — Additional file 1. Percentage of public health facilities that reported malaria indicator data values to the District Health Information Software 2 for all 12 months each year in Kenya, 2011–2015. [file 12936_2017_1973_MOESM1_ESM.docx]

Additional file 1: Percentage of public health facilities that reported malaria indicator data values through the District Health Information System 2 for all 12 months each year in Kenya, 2011–2015

| **Malaria indicators** | **Percentage of health facilities that reported indicator data values for all 12 months by year** | | | | | **% point change** | ***p-*value** |
| --- | --- | --- | --- | --- | --- | --- | --- |
| ***Malaria cases*** | 2011 | 2012 | 2013 | 2014 | 2015 |  |  |
| Confirmed malaria (<5 years) | 7.7 | 10.5 | 11.0 | 11.9 | 15.9 | 8.2 | 0.0001 |
| Confirmed malaria (≥5 years) | 9.9 | 13.7 | 15.2 | 16.0 | 21.1 | 11.2 | 0.0001 |
| Clinical malaria (<5 years) | 24.5 | 30.1 | 17.4 | 12.8 | 5.5 | -19.0 | 0.0001 |
| Clinical malaria (≥5 years) | 24.8 | 32.2 | 19.8 | 15.1 | 7.2 | -17.6 | 0.0001 |
| ***Artemether-lumefantrine treatments*** |  |  |  |  |  |  |  |
| AL weight band 5-14 kg |  | 13.3 | 6.4 | 4.9 | 7.5 | -5.8 | 0.0001 |
| AL weight band 15-24 kg |  | 10.8 | 4.9 | 4.0 | 5.2 | -5.6 | 0.0001 |
| AL weight band 25-34 kg |  | 7.5 | 2.8 | 2.8 | 5.5 | -2.0 | 0.0001 |
| AL weight band ≥35 kg |  | 12.6 | 6.2 | 4.3 | 6.7 | -5.9 | 0.0001 |
| ***Antenatal care and IPTp*** |  |  |  |  |  |  |  |
| New ANC clients | 26.5 | 35.5 | 36.9 | 41.7 | 43.2 | 16.7 | 0.0001 |
| IPTp dose 1^a^ | 39.8 | 47.9 | 49.1 | 44.6 | 13.0 | -26.8 | 0.0001 |
| IPTp dose 2^a^ | 40.3 | 46.9 | 49.4 | 43.0 | 14.0 | -26.3 | 0.0001 |
| ***Long-lasting insecticidal bed nets^b^*** |  |  |  |  |  |  |  |
| LLINs via ANC | 16.1 | 9.3 | 19.2 | 19.7 | 26.0 | 9.3 | 0.0001 |
| LLINs via child health clinics | 2.7 | 4.8 | 10.2 | 9.0 | 9.0 | 6.3 | 0.0001 |
| ***Diagnostic test indicators ^c^*** |  |  |  |  |  |  |  |
| Blood slide tested (<5 years) |  |  |  |  | 1.0 |  |  |
| Blood slide positive (<5 years) |  |  |  |  | 0.5 |  |  |
| Blood slide tested (≥5 years) |  |  |  |  | 1.1 |  |  |
| Blood slide positive (≥5 years) |  |  |  |  | 0.6 |  |  |
| RDT tested^d^ |  |  |  |  | 0.3 |  |  |
| RDT positive^d^ |  |  |  |  | 0.2 |  |  |

AL=artemether-lumefantrine; ANC=antenatal care; IPTp=intermittent preventive treatment in pregnancy with sulfadoxine-pyrimethamine; LLINs=long-lasting insecticidal bed nets; RDT=rapid diagnostic test

^a^Analysis restricted to 1,490 health facilities in the lake-and coast-endemic zones where the IPTp intervention was targeted.

^b^ Analysis was restricted to 4,793 health facilities in 36 counties targeted for routine distribution of LLINs.

^c^ Data only available for 2015.

^d^RDT data not reported by age category.
